# The SAFE procedure: a practical stopping heuristic for active learning-based screening in systematic reviews and meta-analyses

**DOI:** 10.1186/s13643-024-02502-7

**Published:** 2024-03-01

**Authors:** Josien Boetje, Rens van de Schoot

**Affiliations:** 1 Research Group Digital Ethics, Knowledge Center Learning and Innovation (LENI), Archimedes Institute, HU University of Applied Sciences Utrecht, Utrecht, the Netherlands; 2 Department of Methodology and Statistics, Faculty of Social and Behavioral Sciences, Utrecht University, Utrecht, The Netherlands

**Keywords:** Systematic review, Methodology, Active learning, Machine learning, Stopping heuristic, Stopping rule, Meta-analysis, Screening prioritization

## Abstract

Active learning has become an increasingly popular method for screening large amounts of data in systematic reviews and meta-analyses. The active learning process continually improves its predictions on the remaining unlabeled records, with the goal of identifying all relevant records as early as possible. However, determining the optimal point at which to stop the active learning process is a challenge. The cost of additional labeling of records by the reviewer must be balanced against the cost of erroneous exclusions. This paper introduces the SAFE procedure, a practical and conservative set of stopping heuristics that offers a clear guideline for determining when to end the active learning process in screening software like ASReview. The eclectic mix of stopping heuristics helps to minimize the risk of missing relevant papers in the screening process. The proposed stopping heuristic balances the costs of continued screening with the risk of missing relevant records, providing a practical solution for reviewers to make informed decisions on when to stop screening. Although active learning can significantly enhance the quality and efficiency of screening, this method may be more applicable to certain types of datasets and problems. Ultimately, the decision to stop the active learning process depends on careful consideration of the trade-off between the costs of additional record labeling against the potential errors of the current model for the specific dataset and context.

**Supplementary Information:**

The online version contains supplementary material available at 10.1186/s13643-024-02502-7.

## Introduction

Conducting a systematic review or meta-analysis requires a significant amount of time. However, automation can be used to accelerate several steps in the process, particularly the screening phase [1, 13, 16, 23, 26, 31, 33, 34, 36, 43, 45, 48, 51]. Artificial intelligence can assist reviewers with screening prioritization through active learning, a specific implementation of machine learning,for a detailed introduction, we refer to Settles [37]. Active learning is an iterative process in which the machine continually reassesses unscreened records for relevance, and the human screener provides labels to the most likely relevant records. As the machine receives more labeled data, it can use this new information to improve its predictions on the remaining unlabeled records, with the goal of identifying all relevant records as early as possible.

Central to the application of active learning in screening is the objective to screen fewer records than random screening, thereby highlighting the importance of determining an efficient stopping point in the active learning process [55]. However, defining a stopping rule is difficult as the cost of labeling additional records must be balanced against the cost of errors made by the model [15]. Active learning models continually improve their predictions as they receive more labeled data, but the process of collecting labeled data can be time-consuming and resource-intensive. While finding all relevant records is nearly impossible, even for human screeners [52], it is essential to consider that in the absence of labeled data, the number of remaining relevant records is unknown. Therefore, researchers may either stop too early and risk missing essential records or continue for too long and incur unnecessary additional reading [54]. At some point in the active learning process, most, if not all, relevant records have been presented to the screener, and only irrelevant records remain. Thus, finding an optimal stopping point is crucial to conserve resources and ensure the accuracy of the review.

Several statistical stopping metrics have been proposed in the literature [15, 21, 22, 35, 38, 49, 50, 53, 55]). The number of records to screen is based on an estimate of the total number of relevant records in the starting set. For example, randomly screening a predefined set of records and using the observed fraction of relevant records to extrapolate an estimate of relevant records for the complete set [46]. However, these metrics can be difficult to interpret and apply by non-specialists and have not been widely implemented in software tools.

Alternatively, heuristics have been proposed as a practical and effective way to define stopping rules (e.g., [7, 27, 35, 47]). With the time-based approach, the screener stops after a pre-determined amount of time, for example, 1 week. This method can be useful when there is limited screen time or when the screener’s hourly costs are high. With the number-based approach, the screener stops after evaluating a fixed number of records, for example, screening 1 K records. With the data-driven approach, the screener stops after labeling a pre-determined number of consecutive irrelevant records in a row. Lastly, with the key paper heuristic, a set of important papers is determined beforehand, for example, by expert consensus, and the screener stops if all these papers are found with active learning. This method is often used for validating the search strategy by ensuring that the search process adequately identifies relevant primary studies [8, 42].

These single-aspect heuristics offer practical and simple approaches to defining stopping rules for active learning-based screening and can help non-specialists interpret the results more easily. At the same time, using a single heuristic may result in missing potentially relevant records. Therefore,

the goal of the current paper is to present a practical and conservative stopping heuristic that combines different heuristics to avoid stopping too early and potentially missing relevant records during screening. The proposed stopping heuristic balances the costs of continued screening with the risk of missing relevant records, providing a practical solution for reviewers to make informed decisions on when to stop screening. The proposed stopping heuristic is easy to implement and can be effectively applied in various scenarios. The SAFE procedure consists of four phases:

1. Screen a random set for training data;
2. Apply active learning;
3. Find more relevant records with a different model;
4. Evaluate quality.

We first present the results of an expert meeting in which we piloted and discussed the stopping heuristic. Next, we explain the heuristic, including its implementation and effectiveness in different scenarios. Lastly, we discuss the limitations of the proposed method, and we call for future research and adjustments to the method to make it fit different scenarios.

## Development

### Method

The proposed stopping heuristic was initially developed in December 2022 and was inspired by the procedure used by Brouwer et al. [9]. It was subsequently peer-reviewed on 12–01-2023 by a group of 26 experts comprising information specialists, data scientists, and users of active learning-aided systematic reviews from the Netherlands and Germany. The proposed stopping heuristic was presented to the participants, who provided feedback on several aspects, including the use of a minimum percentage to screen, a conservative standard to determine the pre-determined number of consecutive irrelevant records, the inclusion of a visual inspection of the recall plot as part of the stopping heuristic, and the use of key papers as prior knowledge. The feedback was collected digitally via Wooclap software, discussed by the authors, and used to adapt the stopping heuristic accordingly, resulting in a practical and effective stopping rule that can be implemented in systematic reviews and meta-analyses using active learning.

### Results

First, the participants of the expert meeting were very enthusiastic about the general setup of the proposed stopping heuristic and agreed with the different stages, as they felt that it was a practical and effective solution to determine when to stop screening in systematic reviews and meta-analyses using active learning. They appreciated the conservative and practical approach, which would help ensure that relevant records are not missed while minimizing the amount of unnecessary screening.

The participants were in favor of using a minimum percentage of records to screen but emphasized the importance of linking it to an estimate of the fraction of relevant records in the total dataset to avoid stopping too early. As one colleague noted, “Yes, I'd opt for a minimum percentage. I'd decide on this percentage based on the initial screening that you do. That percentage can be used as an indication of what percentage of articles will be relevant in the total sample.”. However, since this percentage could be either a under-or overestimation of the actual fraction of relevant records, colleagues advised using a minimum percentage based on simulation studies using active learning and building in a margin for the irrelevant records that may be incorrectly marked. This approach would help ensure a conservative stopping rule that balances the costs of continued screening with the risk of missing relevant records.

Second, researchers were positive about using a visual inspection of the recall plot (“Incredible idea, promising!”, “Seems very logical and rational to me”), but they considered it to be not precise enough as a stopping rule on its own (“With other rules combined it is good enough”, “I think it works if you have other stopping rules (such as the minimum %”, “I think it may be best to combine a percentage range and this”). However, visual inspection of the recall plot can be used to get an indication of whether it's time to apply the stopping heuristic. This makes the screening process more efficient, as applying the stopping rule(s) takes valuable time (e.g., checking for key papers).

Researchers shared their experience and agreed that a combination of the minimum percentage of records screened and a threshold of 50 consecutive irrelevant records was a “safe and reasonable” approach. The combination of these two checks helps minimize the risk of screening an excessive number of irrelevant records while ensuring enough relevant records are included in the review process. However, the experts acknowledged that a higher number of consecutive irrelevant records might be necessary for some applications, for instance, where labeling time is inexpensive, or where it is crucial to identify as many relevant records as possible. It is important to note that humans typically miss around 10% of the relevant records [52], and some relevant records may not be included in the dataset due to limitations in the search or errors in the metadata of records.

During the expert meeting, it was agreed that using key papers to check screening results was a good practice. However, the researchers reached a consensus that these papers might not be the best set to use as prior knowledge in active learning, as they could be biased by the method used to identify them. For instance, experts asked to provide key papers in their field might be biased towards citing papers from their colleagues, which may not represent the relevant papers in the total dataset. Therefore, incorporating key papers as prior knowledge in active learning could result in a biased model. Nevertheless, key papers can still be used to validate the stopping heuristic. The input from the peer-review session led to the formulation of the SAFE procedure containing a set of stopping heuristics.

## The SAFE procedure

### Assumptions

The proposed procedure is meant to determine when to stop screening when applying active learning-aided screening while adhering to the PRISMA 2020 statement [29] and Open Science principles to ensure reproducibility and transparency for AI-aided output [24]. It is designed to be conservative and easily understood by non-experts and to enable finding a reasonable percentage of relevant records in the dataset rather than aiming for 100% [8, 30]. The procedure can be applied to the title/abstract screening phase, but it can also be combined with the full-text screening phase (see, for example, [9]). To achieve optimal results, we expect users to input high-quality data with minimal missing titles or abstracts and as few duplicates as possible, adhering to the “garbage in, garbage out” (GIGO) principle.

The method further assumes a set of key papers from the field that should be included in the final selection. Also, it is expected two screeners will independently screen the records, as advised by the PRISMA 2020 recommendations [29]. Any disagreements should be solved before starting the next phase.

### The four phases

**Fig. 1:**
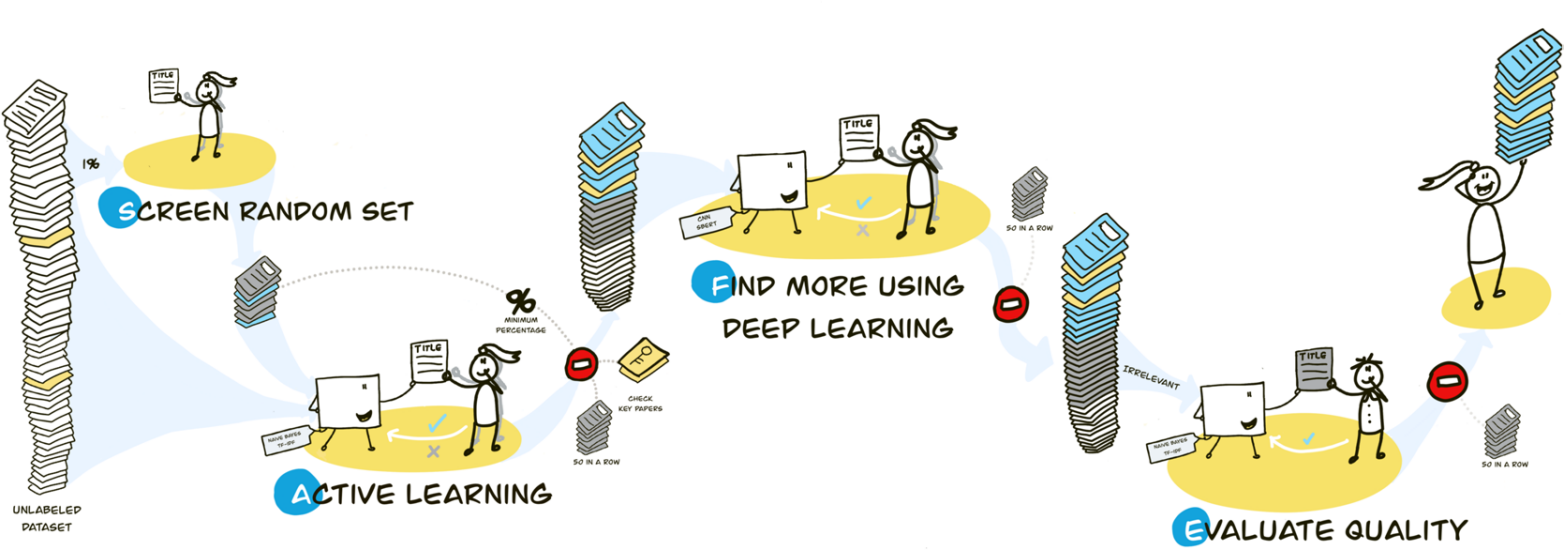
Graphical overview of the SAFE procedure for applying a practical stopping heuristic for active learning-aided systematic reviewing [4] Note: Disclaimer: The numbers provided in this figure are arbitrary and should not be considered universally applicable. Researchers are responsible for choosing appropriate values based on their specific situation and requirements. The SAFE procedure is a flexible framework, and its effectiveness depends on the careful selection of parameters tailored to the context of each systematic review

The SAFE procedure consists of four phases and is graphically displayed in Fig. 1. For practical guidance on implementing the SAFE procedure, we have included a comprehensive ‘cheat sheet’ [see Additional file 1]. This adaptable resource provides a framework for researchers to input their chosen machine learning models and parameters, along with stopping heuristics, tailored to the specific needs of their review. Note that we provide some values, like percentages or the number of records; these numbers should be merely used as an example and are in no way meant as exact rules. Similarly, the machine learning models referenced here serve as examples; researchers should feel encouraged to select or adapt models that best suit their specific requirements and preferences.

#### Phase 1: screen a random set of training data

In order to train the first iteration of the machine learning model, it is necessary to have training data available consisting of at least one labeled relevant record and one labeled irrelevant record. While key papers could be used for this purpose, the expert meeting suggested that such papers might introduce bias. Therefore, we propose to start by labeling a random set of records for the training data (e.g., 1% of the total number of records). The advantage of not using the key papers is that this phase can also be used for the calibration of the inclusion/exclusion criteria and training of the screeners. We propose to use a stratified selection of records, deliberately incorporating subsets from the spectrum of publication dates: the oldest, the newest, and a random sampling from intermediate periods. This approach ensures a representative coverage of the evolving meanings and interpretations of scientific concepts over time, which will help enrich the content of the training data.

The stopping rule for this phase is to screen a minimum of records or up to the point where at least one relevant record is found.

Based on the results, the Fraction of Relevant Records in the training set (FRR_t) can be calculated by dividing the number of Relevant Records in the training set (RR_t) by the total number of records in the training set (t). If the records in the training set are a random subset, multiplying the FRR_t by the total number of records T provides a crude estimate of the number of Relevant Records in the total dataset (RR_T), which will be used in the stopping heuristic of the second phase to provide a rough minimum of records to be screened. Note that much better estimation techniques are available (e.g., [46]), and we return to this issue in the discussion section.

#### Phase 2: apply active learning

The second phase concerns screening via active learning aiming to find all or as many relevant records as possible with minimal screening effort. The first iteration of the active learning model, for example, Naive Bayes or logistic regression as the classifier and TF-IDF as the feature extractor, will be trained using the labeled dataset from Phase 1. The model chosen should be computationally cheap and should be shown to be efficient in several simulation studies.

During the active learning phase, the stopping heuristic is a four-fold rule: screening will be stopped when all of the following four mutual independent conditions are met:

- All key papers have been marked as relevant;
- At least twice the RR_T records have been screened;
- A minimum of 10% of the total dataset has been screened;
- No relevant records have been identified in the last, for example, 50 records.

**Fig. 2:**
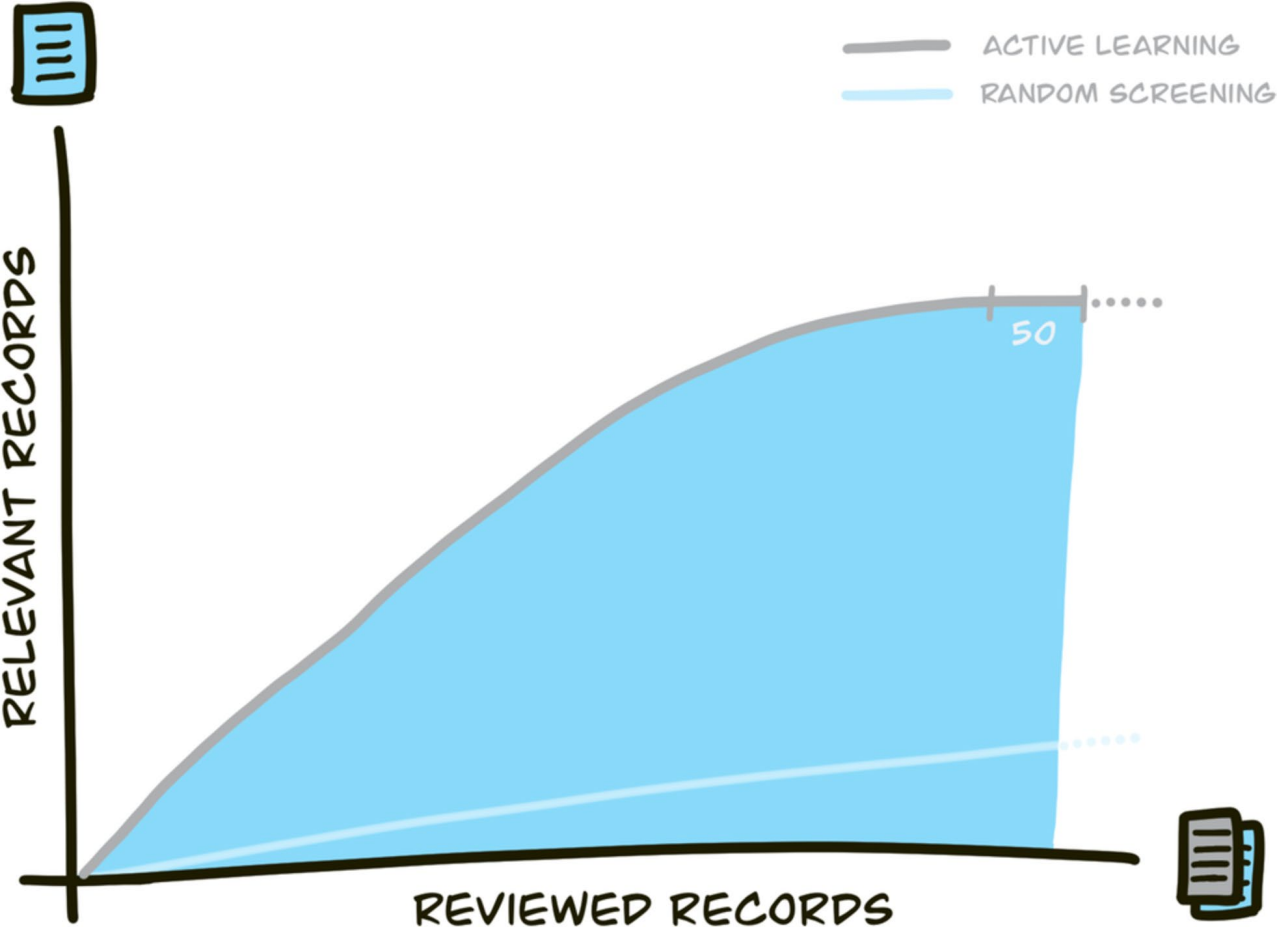
Example recall plot comparing the number of identified relevant records against the number of viewed records for active learning (grey line) versus random screening (blue line) [5]

During the active learning phase, it may be helpful to inspect the recall plot in instances where a large number of consecutive records have been marked as irrelevant. The recall plot shows the number of identified relevant records against the number of viewed records. A visual analysis of the plot can reveal whether a plateau has been reached (see Fig. 2), indicating that the probability of identifying new relevant records has become small. Once this plateau has been visually identified, the remaining stopping rules can be checked (e.g., check if the key papers already have been found) to determine whether it is appropriate to halt the screening process for this phase.

#### Phase 3: find more relevant records with a different model

The third screening phase is to ensure that records are not missed due to suboptimal choice of the active learning model [40]. It might be that some relevant records are not presented to the reviewer because the text used in the abstract is not seen as potentially relevant because of concept ambiguity [11, 17], which can make finding relevant records challenging. To identify such records, the algorithms must “dig deeper” into a text to find its essence [18]. This problem is best tackled with deep learning models, which are better at finding complex connections within data than shallow networks like the simple model used in the first screening phase. However, deep learning models require more training data [2] and are not expected to perform well in the first few iterations [39]. Hence, the labeling decisions from the first screening phase will be used as prior knowledge to train a different model, for example a neural network model as a classifier along with sBert as the feature extractor. The unlabeled records will be re-ordered using this different algorithm, and screening can continue to check if the first model has missed relevant records. The stopping rule for the third screening phase dictates that screening will stop if no extra relevant records are identified in the last, for example, 50 records.

#### Phase 4: evaluate quality

Quality checks are an essential part of a systematic review to ensure that the systematic review is as comprehensive and accurate as possible. Therefore, in Phase 4, the goal is to identify any excluded but relevant records from the previous phases. The records that were previously labeled as irrelevant will be screened using a simple model, for example, Naive Bayes as the classifier and TF-IDF as the feature extractor. To train the active learning model, the 10 highest- and lowest-ranked records from the previous phase can be used. An independent screener can then go through the most likely to be relevant but excluded records to identify any relevant records that might have been excluded. The screening process will continue until the stopping rule is met, which is when no extra relevant records are identified in the last, for example, 50 records (see for an application Neeleman et al. 2023).

To ensure the comprehensiveness of the systematic review, additional quality checks can be performed using forward and/or backward citations with the final inclusions. This method is also suggested by the SYMBALS methodology [46]. This can be automated, for example, through the use of SR-Accelerator’s Spidercite [14], Citation Chaser [19]. Additionally, as an extra quality check, the complete author team can go through the records identified as relevant to check for incorrectly included but irrelevant records based on the inclusion criteria. Any irrelevant records will be marked and removed from the dataset of relevant records. This extra quality check can help ensure the accuracy and reliability of the final results. 

### Time investment in the four phases

**Fig. 3:**
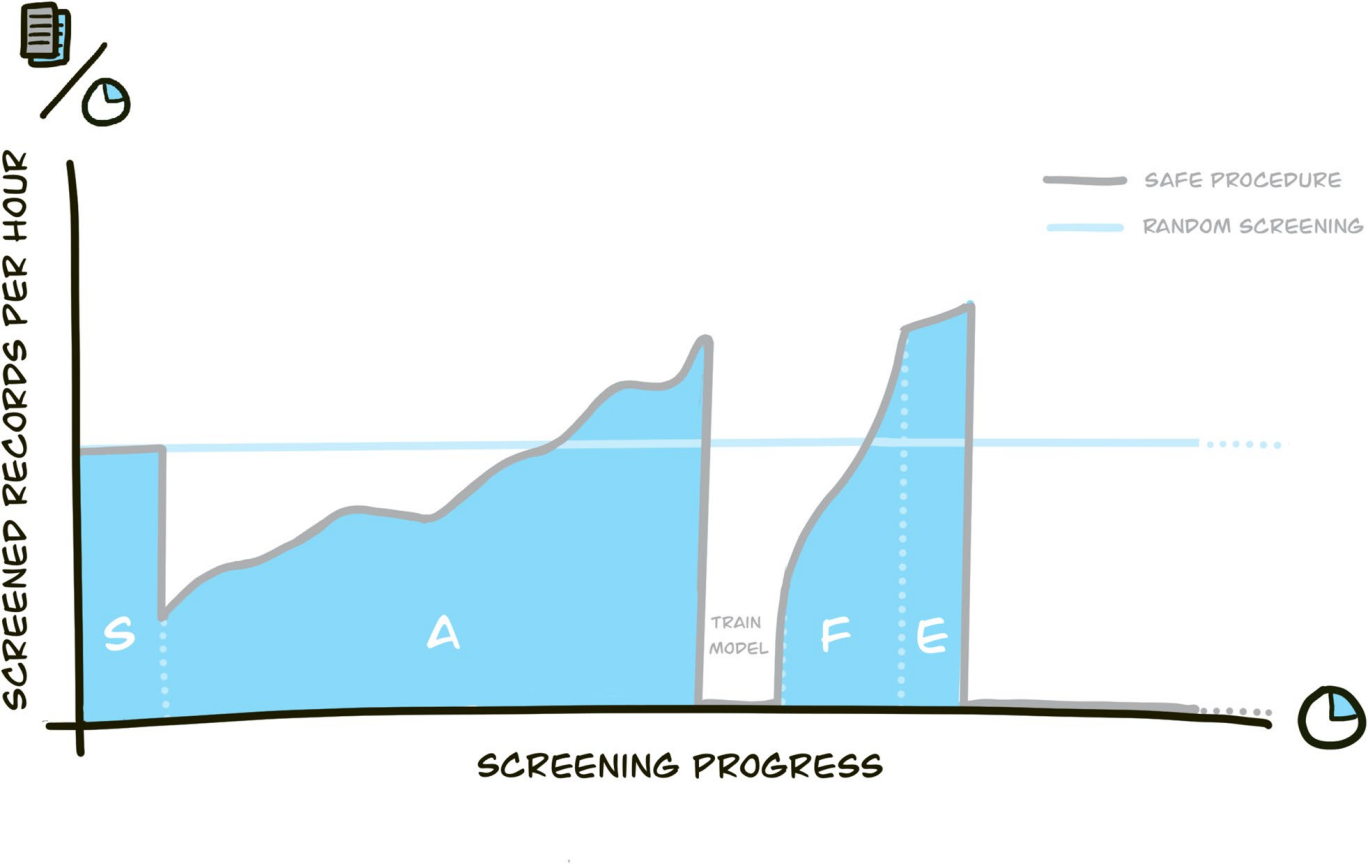
Screening speed over time compared between active learning using the SAFE procedure and random screening [6]

Since the goal is to save time by using active learning, an estimate of the screening speed and relative duration of the four phases is displayed in Fig. 3. The time investment for the first phase is equal to that of random screening. At the beginning of the second phase, it is expected that more time will be needed to screen for relevance. This is because the active learning model puts the most likely relevant records upfront. However, during the early screening phase of the second screening phase, it is also expected that the more challenging records will be presented, which may require discussion on how to apply the inclusion and exclusion criteria exactly. In the third phase, the training of the deep learning model may require significant computation time, depending on the dataset’s size and the neural network’s complexity. Table 1 in Teijema et al. [39] provides expected training times for neural networks: up to 6 h on a high-performance cluster. Again, the first set of records presented might be challenging, but soon, obviously, irrelevant papers will be presented. The fourth phase is relatively quick, taking maybe only 1–2 h to complete. After the fourth phase, the records that are most likely not relevant will not be seen by the screener, thus saving time when compared to random screening.

Overall, the SAFE procedure significantly speeds up the screening process and increases the efficiency of the review. However, it is important to note that the time invested in the screening process may vary depending on the complexity of the dataset and the specific active learning model used.

### Software

Priority screening via active learning has been successfully implemented in various software tools such as Abstrackr [49], ASReview [45], Colandr [12], EPPI-Reviewer [41], FASTREAD [54], Rayyan [28], RobotAnalyst [32], Research Screener [10], DistillerSR [20], and robotreviewer [25]. For a curated comparison of these software tools, see van de Schoot [44]. Among these tools, only the free and open-source software ASReview LAB [3] offers the flexibility to implement the suggested model-switching approach proposed in this paper.

## Discussion

The utilization of active learning in systematic reviews is gaining more attention as it can improve the accuracy of the screening process and save time. However, there is a risk of missing relevant records if the screening process stops too early. In this paper, we have presented a procedure with stopping heuristics, aiming to balance time efficiency and completeness of the screening. The SAFE procedure includes a preliminary screening phase to warm up the AI model, an active learning phase to find as many relevant records as possible, model switching to ensure that records are not missed due to suboptimal choice of the active learning model, and a quality check phase to avoid incorrectly excluding relevant records. We believe that our procedure can provide valuable guidance for researchers and practitioners in the field of systematic reviews who want to use active learning to improve their screening process.

In addition to the proposed set of stopping heuristics, many other potential stopping rules are described in the literature. These include computing an inflection point [16, 21, 35, 38, 49, 50], estimating recall for the sequential screening of a ranked list of references [22], or computing the lengths of consecutive spans of excluded documents that occur between each relevant document during screening [38, 53, 55]. It is important to note that these methods can also be used for the task at hand: to determine when to stop the active learning process. Their suitability should be evaluated for each specific problem and dataset. We decided not to rely on such methods because they require advanced statistical knowledge, making them less accessible to non-expert reviewers. As such, it is essential to carefully evaluate each stopping rule’s potential benefits and drawbacks and choose the most appropriate one based on the specific context, expertise, and resources available.

We acknowledge that a direct comparison with existing stopping heuristics has not been presented in this paper. However, our proposed SAFE procedure aims to provide a comprehensive and effective solution by combining an eclectic mix of stopping heuristics to minimize the risk of missing relevant papers. This unique approach sets it apart from other methods that typically focus on only one aspect of the screening process. In future research, a comparative analysis of the SAFE procedure with other established heuristics could further validate its effectiveness and efficiency in different contexts and settings.

It is worth noting that while active learning can significantly improve the efficiency and quality of the screening process, it also has its limitations. As the machine receives more labeled data, it can improve its predictions, but there may be a point of diminishing returns in terms of computation time and resources. This is particularly true for the deep learning phase, which may require extensive training times, especially for large datasets of over 50,000 records [39]. Cloud computing can help optimize processing times but may not always be practical or feasible. Moreover, it is important to note that by the time a researcher arrives at phase three, most, if not all, of the relevant records may have already been identified. Therefore, the trade-off between the additional training and screening time required during the deep learning phase and the potential gains of identifying a few more relevant records should be carefully considered. It is important to note that during this phase, the model may identify records with slightly different textual structures, which may or may not be relevant to the review. Of course, it also depends on the availability of selecting different models in the software and options to run the software in the cloud. Ultimately, the decision of whether to invest in additional training and screening time in this phase should be based on a careful consideration of the potential gains and the costs involved, including the time and resources required to train the model and the potential impact of the additional records on the review's conclusions.

Another consideration is the cut-off values we used as an example. While the heuristic of using twice the observed fraction of relevant records in a preliminary set of 1% is a useful rule of thumb, it may not always be suitable for small datasets. For example, when working with a dataset of only 500 records, screening 1% would mean only 5 records are screened, and the observed fraction in such a small sample may not yield a representative estimate of relevant records for the complete set. Whereas the minimum of 1 relevant record limits the risk of underestimation, this method could easily lead to an overestimation of the fraction of relevant records in the complete set. In these cases, the rule of thumb could lead to unnecessarily screening too many records, but at the same time makes the SAFE procedure more conservative. Researchers should exercise caution and use appropriate statistical methods to estimate the fraction of relevant records when working with small datasets. When resources are not an issue, in some cases, it might be equally suitable to screen the whole set manually.

Furthermore, the proposed batch size for the number of irrelevant records in a row depends on the research question, the domain of the review, and the desired level of recall. For example, in the field of medicine, missing any relevant records might not be acceptable, so a larger batch size is advised. Nevertheless, it is crucial to balance the cost of labeling more records with the cost of errors made by the current model and choose appropriate stopping rules to achieve the desired level of recall.

While active learning can significantly improve the efficiency and quality of the screening process, its application requires careful consideration of its applicability to specific review types and datasets. Further research should aim to tailor these heuristics to diverse settings and needs. For example, the procedure is suitable for updating systematic reviews or conducting living systematic reviews. The already labeled records from the initial review can be used for training data in Phase 2. Adapting the general heuristics to specific settings could increase the ease of applying the SAFE procedure for researchers across various disciplines and contexts. However, the proposed procedure may not be applicable to all active learning scenarios, as they may only apply to specific types of data and models.

In conclusion, this paper introduces a structured procedure for using active learning in the screening phase of a systematic review, consisting of four phases with their stopping heuristics. It presents a systematic approach, balancing the costs of additional labeling against the risk of model errors, to inform the decision on when to stop the active learning process while screening. Overall, the proposed procedure provides a practical, conservative, and efficient solution for determining when to stop with active learning in the screening phase of systematic reviews, which non-experts in the field can easily implement.

### Supplementary Information

**Additional file 1. **SAFE Procedure Cheat Sheet. Description of data: This'cheat sheet' serves as a practical guide for the SAFE procedure. This adaptable resource provides a framework for researchers to input their chosen machine learning models and parameters, along with stopping heuristics, tailored to the specific needs of their review.

## Data Availability

Not applicable.
