# Supplementary material for: The SAFE procedure: a practical stopping heuristic for active learning-based screening in systematic reviews and meta-analyses
Source: Syst Rev. 2024 Mar 1;13:81. doi: 10.1186/s13643-024-02502-7 (PMC10908130; doi:10.1186/s13643-024-02502-7)
Supplement: Supplementary file 1 — Additional file 1. SAFE Procedure Cheat Sheet. Description of data: This'cheat sheet' serves as a practical guide for the SAFE procedure. This adaptable resource provides a framework for researchers to input their chosen machine learning models and parameters, along with stopping heuristics, tailored to the specific needs of their review. [file 13643_2024_2502_MOESM1_ESM.pdf]

Custom SAFE procedure Cheat Sheet

Fill in the blanks with your own models and parameters tailored to your review.

	DATASET	MODEL	PRIOR KNOWLEDGE	STOPPING HEURISTIC(S)
**SCREEN RANDOM SET**<br>Partly label the dataset to warm-up the AI model;<br>Get an indication of the fraction of relevant records (FRR_T)	Random subset	-	-	• Screen a minimum of _% of the total number of papers;<br>• Find at least one relevant record.
**ACTIVE LEARNING**<br>Find as many relevant records as possible	Complete dataset	Simple model<br>classifier: ____________<br>feature extractor: ____________	Labeled records phase 1	• All key papers have been marked as relevant;<br>• At least twice the estimate of the number of relevant records (___ records) have been screened;<br>• A minimum of __% of the total dataset has been screened.<br>• No extra relevant records have been identified in the last __ records.
**FIND MORE USING DEEP LEARNING**<br>Ensure that records are not missed due to suboptimal choice of the active learning model	Complete dataset	Deep learning model<br>classifier: ____________<br>feature extractor: ____________	Labeled records phase 1 and 2	• No extra relevant records are identified in the last __ records.
**EVALUATE QUALITY**<br>Avoid incorrectly excluded relevant records	All records labeled as irrelevant	Simple model<br>(see phase 2)	The 10 highest- and lowest-ranked papers from phase 2	• No extra relevant records are identified in the last __ records.

The artwork of the logos is by Joukje Willemsen: https://github.com/asreview/asreview-artwork (CC BY-NC-ND 4.0)
